# 

**Published:** 1886-06

**Authors:** 


					﻿JTTNE, 1386-
X\\BRAft Y \
(indexed
S.G.O.
•OUR - ADDITIONAL PAGES.
OLD SERIES
VoJ XXV
<7 I85S _O"
NEW SERIES
Vol. III.-Nq.4.__
HEDIGIHjW®
MB TmmmieSsn
tflUUnlNAu^ti

V £

y W.F. WESTMOREL AND. NOB <
H.V.IMMILUJE.M.DLL. jJO
JAMES A.GRAY. MLL^
MfflJSHED By lames p.h/arissn&.c(C|
if Atlanta.ga. taBL
SUBSCRIPTION' 12.00 A YEAR.
				

